# Thoracoscopic and hand assisted laparoscopic esophagectomy with radical lymph node dissection for esophageal squamous cell carcinoma in the left lateral decubitus position: a single center retrospective analysis of 654 patients

**DOI:** 10.1186/s12885-017-3743-1

**Published:** 2017-11-10

**Authors:** Masahiko Murakami, Koji Otsuka, Satoru Goto, Tomotake Ariyoshi, Takeshi Yamashita, Takeshi Aoki

**Affiliations:** Department of Surgery, Division of Gastroenterological and General Surgery, School of Medicine, Showa University, 142-8666, 1-5-8 Hatanodai, Shinagawa-ku, Tokyo, Japan

**Keywords:** Carcinoma of the esophagus, Thoracoscopy, Left lateral decubitus

## Abstract

**Background:**

The rates of thoracoscopic esophagectomy performed in the prone and left lateral decubitus positions are similar in Japan. We retrospectively reviewed short- and long-term outcomes of thoracoscopic esophagectomy for esophageal cancer performed in the left lateral decubitus position.

**Methods:**

Between 1996 and 2015, 654 patients with esophageal cancer underwent thoracoscopic esophagectomy in the left lateral decubitus position. Patients were divided into early (1996–2008) and late groups (2009–2015, with standardization of the procedure and formalized training), and their clinical outcomes reviewed.

**Results:**

The completion rate of thoracoscopic esophagectomy was 99.5%, and the procedure was converted to thoracotomy in three patients, due to hemorrhage. The mean intrathoracic operative time, intrathoracic blood loss, and number of dissected mediastinal lymph nodes were 205.0 min, 127.3 mL, and 24.7, respectively. Postoperative complications included pneumonia (8.5%), anastomotic leakage (7.5%), and recurrent nerve paralysis (3.5%). Postoperative (30d) mortality was 4/654 (0.61%) due to anastomotic leak and pneumonia. The five year overall survival rate was 70%. A comparison of the 289 early- and 365 late-study period cases revealed significant differences in mean intrathoracic blood loss (174.0 vs. 94.2 mL), number of mediastinal lymph nodes dissected (20.0 vs. 28.4), hospital length of stay (33.4 vs. 20.0 days, *p* < 0.001), and postoperative anastomotic leakage (14% vs. 1.6%, *p* < 0.0001).

**Conclusions:**

Standardization of the procedure for thoracoscopic esophagectomy in the left lateral decubitus position, with a standardized clinical pathway for perioperative care led to significant improvements in surgical outcomes.

## Background

In Japan, thoracotomy with complete lymph node dissection in the cervical, mediastinal, and abdominal regions has been performed for esophageal cancer since the 1980s with favorable outcomes.[1–3] However, this procedure is invasive and can result in a high incidence of complications, particularly pulmonary complications. [4] Mouret reported the first laparoscopic cholecystectomy in 1987, and this surgical approach has subsequently been applied to a wide range of organs and diseases. Cushieri et al. initially reported performing thoracoscopic resection of esophageal cancer in 1992, [5] and many groups have since shown its utility, including Akaishi et al., [6] Kawahara et al., [7] and Ohsugi et al. [8] in Japan.

Palanivelu et al. first described thoracoscopic esophagectomy in the prone position in 2006, [9] and many surgeons in Japan perform the operation with the patient in this position, [10, 11] with a similar number of resections performed in the left lateral decubitus position. We began performing complete thoracoscopic esophagectomy in the left lateral decubitus position in 1996, and from November 1996 to July 2015, performed 654 procedures using this approach. This is a review of patients who underwent thoracoscopic esophagectomy in the left lateral decubitus position in a single hospital. All operations were performed by three surgeons. The procedure has been adapted and modified, and finally the procedure and perioperative protocol were standardized in January 2009. In this study, we investigated the short- and long-term outcomes of these 654 patents with esophageal cancer treated with thoracoscopic resection in the left lateral decubitus position over the last 20 years and compared early (1996–2008) and late (2009–2015) study periods.

## Methods

Between 1996 and the first half of 2015, thoracoscopic resection for patients with esophageal cancer in the left lateral decubitus position was attempted in 654 patients at Showa University Hospital. This includes all patients with carcinoma of the esophagus seen in our institution during the study period, except for three patients who underwent thoracoscopic esophagectomy in the prone position and 30 patients who underwent mediastinoscopic esophagectomy. Three procedures were converted to open thoracotomy due to complications, for a thoracoscopic completion rate with patients in the left lateral decubitus position of 99% (651/654). Surgical indications included patients with carcinoma of the thoracic esophagus, without serious heart or respiratory disease that would preclude safe conduct of surgery under general anesthesia, without metastases to other organs such as lung or liver, and tumor stage lower than Stage T4b. No specific age restriction was established; the oldest patient was 93 years of age. Patients treated preoperatively with chemotherapy or chemoradiotherapy are included in this review. Clinicopathological factors were classified according to UICC-TNM (7th edition) criteria, [12] and complications investigated using the Clavien-Dindo classification. [13] Outcomes and complications were compared between patients treated in the early (1996–2008) and late (2009–2015) periods. In the late period, the procedure was standardized, and surgeon training was formalized.

### Statistical analysis

Summary statistics were presented by medians with standard deviation (SD), and number with proportion (%). Each factor was analyzed with Student’s t-test and Fisher’s exact test. Survival curves were prepared using the Kaplan-Meier method and the curves compared by Log-Rank difference (*P*-value) at each pathological stage. Cox hazards analysis was used to assess the association between time period and survival, adjusted by operation time, neo adjuvant therapy (no adjuvant therapy vs. any adjuvant therapy), blood transfusion (no blood transfusion vs. blood transfusion), complications (no complications vs. any complications). These control variables were selected due to their clinical importance.

We calculated odds ratios using logistic regression models to determine factors associated with survival. Control variables based on significant differences in univariate analysis and clinically important factor were selected including age, neo-adjvant therapy, abdominal procedure, reconstruction conduit, reconstruction route, anastomosis site, thoracic blood loss, number of retrieved thoracic lymph nodes, number of retrieved total lymph nodes.

The threshold for statistical significance was *p* < 0.05. All statistical analyses were performed using JMP software ver.13.

### Anesthesia, position, and port arrangement

**Fig. 1:**
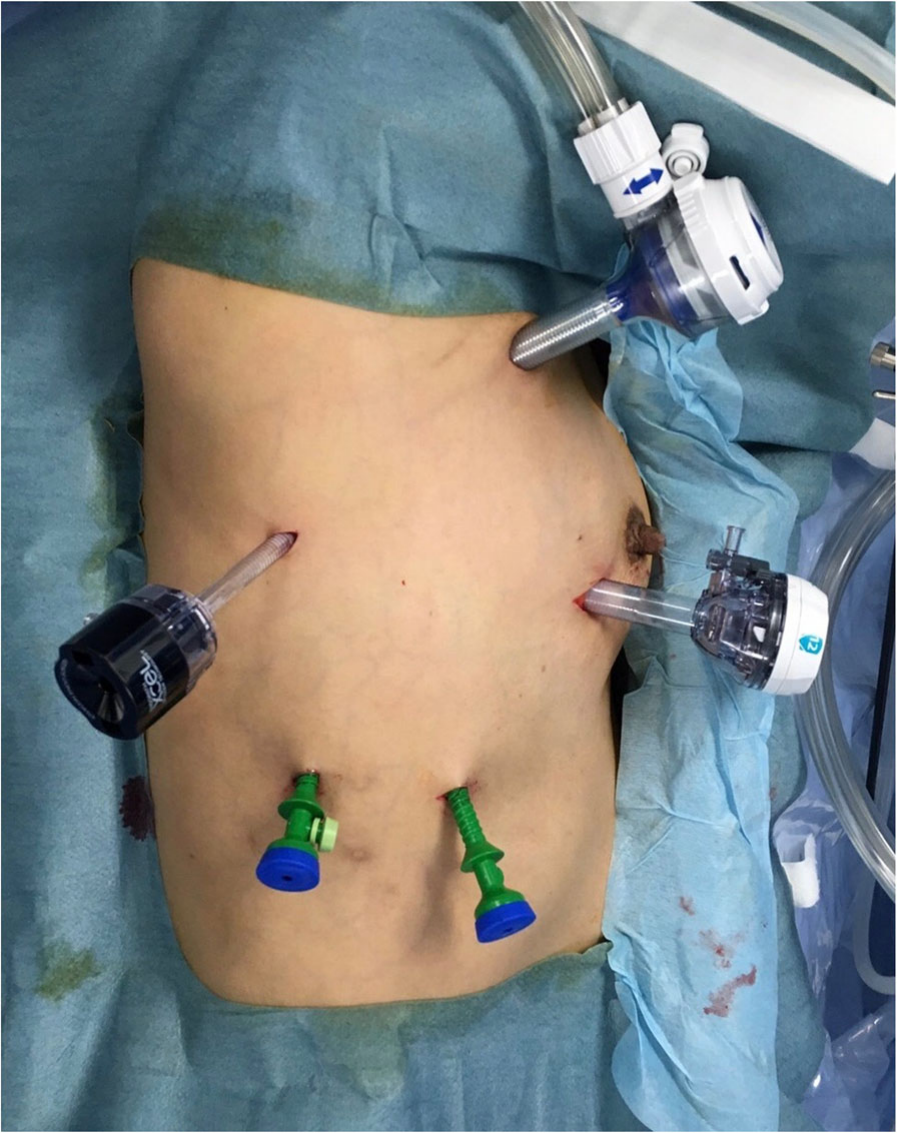
Port placement: Three 5-mm ports and two 12-mm ports are used

Surgery is performed after induction of general anesthesia. One-lung pulmonary ventilation using an 8-Fr spiral tube was used, and a blocker was placed into the tube to block the right mainstem bronchus. The thoracic portion of the operation was performed in the left lateral decubitus position with 15° head elevation and slight rotation of the bed toward the dorsal side. A video monitor was placed at the patient’s head (single-monitor method), and the operator and assistant have the same visual field. As a basic port arrangement, 5-mm ports for the operator were inserted into the 5th and 8th intercostal regions on the posterior axillary line, a 5-mm port for the thoracoscope was inserted into the 8th intercostal region at the middle axillary line, and 12-mm ports for the assistant were inserted into the slightly ventral 3rd intercostal region and 5th intercostal region on the anterior axillary line. Normally, the 5th intercostal port for the assistant’s left hand was inserted first, and after initial exploration, the port positions were adjusted based on the patient’s physique (Fig. 1).

### Thoracic procedure

**Fig. 2:**
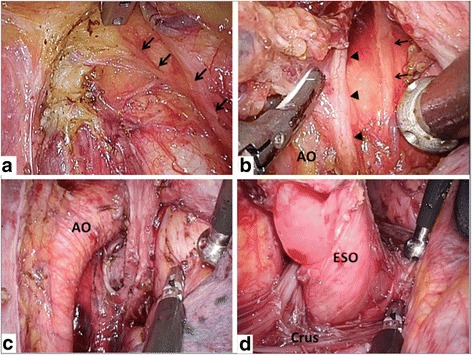
The view after each component of the thoracic dissection. **a** After dissection of the right recurrent laryngeal nerve lymph nodes: the arrow indicates the right recurrent laryngeal nerve. **b** After dissection of the left recurrent laryngeal nerve lymph nodes: arrow indicates the cardiac branch of the sympathetic nerve and the arrowhead indicates the left recurrent laryngeal nerve. **c** After dissection of the subcarinal and main bronchus lymph nodes. **d** After dissection of lower mediastinal lymph nodes. ESO: esophagus, Crus: crus of the diaphragm, AO: aortic arch

The procedure is performed as follows:

1. The lymph nodes around the right recurrent laryngeal nerve are dissected. Since three to four branches run from the right recurrent laryngeal nerve toward the esophagus, these are divided sharply with scissors (Fig. 2a). On the cranial side, the lymph node dissection is advanced to the level of the inferior thyroid artery.
2. After dissection around the upper thoracic esophagus, the esophagus is transected using an automatic suture device (Echelon Gold 60 mm, Johnson and Johnson, New Brunswick NJ USA).
3. The assistant rotates the trachea toward the ventral side, and the lymph nodes around the left recurrent laryngeal nerve are dissected (Fig. 2b).
4. The tracheal bifurcation area lymph nodes are dissected (Fig. 2c).
5. Finally, the middle and inferior mediastinal lymph nodes are dissected including supradiaphragmatic lymph nodes and the dorsal lymph nodes around the thoracic descending aorta (Fig. 2d).
6. After the thoracic portion of the procedure, a 15-Fr J-VAC drain and an 8-Fr aspiration catheter are placed in the thorax. The 15-Fr J-VAC drain is removed the day after surgery if no air leak is apparent, and only the 8-Fr suction catheter is left for drainage.

### Abdominal and cervical procedures

After the thoracic resection, abdominal and cervical operations are performed. In the abdominal portion, the lymph nodes around the stomach are dissected laparoscopically with manual assistance, and the gastric tube prepared. For patients with a history of gastric surgery or concomitant gastric cancer, the right colon was used for reconstruction. Reconstruction was performed through the retrosternal route, and anastomosis performed in the cervical region. Patients who had undergone previous sternotomy (e.g. previous cardiac surgery), were reconstructed using the mediastinal route. The gastric tube was created using a hand-assist technique because we believe that this is more gentle than using laparoscopic instruments, and may lead to less tissue injury and subsequent associated complications. The cervical anastomosis is created with a 25 mm circular stapler using the end of the esophagus into the side of the gastric wall.

Three-region lymph node dissection was performed in the cervical esophageal, upper thoracic, and middle thoracic regions, and two-region dissection was performed in the lower thoracic region and abdominal esophagus.

### Postoperative management

The tracheal tube was removed immediately after surgery. Patients were treated in the intensive care unit on the day after surgery and transferred to a high care unit, started walking and drinking water on day 2, returned to the general surgical ward on postoperative day 3, started eating food on day 5, and discharged on day 9 or later.

## Results

Demographic characteristics for all patients and each study period (early and late) are shown in Table 1. The gender ratio, tumor location, and histopathological diagnosis are comparable to data from other institutions in Japan.14 Preoperative therapy was given to 72% of patients. Lung adhesions were noted during surgery in 32% of patients. Thoracoscopic esophagectomy was attempted in all patients, but the procedure was converted to thoracotomy in three patients (0.5%) in the early period due to hemorrhage (*n* = 2) and damage to the trachea (*n* = 1).

**Table 1:** Patient Demographics- all study patients

	All number (*n* = 654) (%)	Early (*n* = 289)	Late (*n* = 365)	*p*
Age, mean (SD)	64.9	64.9 (9.5)	66.3 (9.0)	0.03
Male, n (%)	539 (82.4)	239 (82.7)	300 (82.2)	
Tumor location				0.01
Cervical, n (%)	14 (2.1)	7 (2.4)	7 (1.9)	
Upper, n (%)	76 (12)	40 (14)	36 (9.9)	
Middle, n (%)	344 (53)	131 (45)	213 (58)	
Lower, n (%)	197 (30)	100 (35)	97 (27)	
Abdominal, n (%)	23 (3.5)	11 (3.8)	12 (3.3)	
Histologic findings				0.05
Squamous cell carcinoma, n (%)	616 (94)	271 (94)	345 (95)	
Adenocarcinoma, n (%)	14 (2.1)	4 (1.4)	10 (2.7)	
Adenosquamous carcinoma, n (%)	7 (1.1)	2 (0.7)	5 (1.4)	
Carcinosarcoma, n (%)	6 (0.9)	1 (0.3)	5 (1.4)	
Basaloid cell carcinoma, n (%)	5 (0.8)	4 (1.4)	1 (0.3)	
Small cell carcinoma, n (%)	4 (0.6)	4 (1.4)	0 (0)	
Neuroendocrine cell carcinoma, n (%)	1 (0.2)	0 (0)	1 (0.3)	
Malignant melanoma, n (%)	1 (0.2)	0 (0)	1 (0.3)	
pTNM Stage				0.00
Stage IA, n (%)	215 (33)	68 (24)	147 (40)	
Stage IB, n (%)	27 (4.1)	12 (4.2)	15 (4.1)	
Stage IIA, n (%)	72 (11)	43 (15)	29 (7.9)	
Stage IIB, n (%)	73 (11)	29 (10)	44 (12)	
Stage IIIA, n (%)	99 (15)	45 (16)	54 (15)	
Stage IIIB, n (%)	57 (8.7)	30 (10)	27 (7.4)	
Stage IIIC, n (%)	49 (7.5)	27 (9.3)	22 (6.0)	
Stage IV, n (%)	62 (9.5)	35 (12)	27 (7.4)	
Neoadjuvant therapy				<0.001
None, n (%)	185 (28)	151 (52)	34 (9.3)	
Chemotherapy, n (%)	337 (52)	39 (13)	298 (82)	
Chemo-radiation, n (%)	129 (20)	97 (34)	32 (8.8)	
Radiation, n (%)	3 (0.5)	2 (0.7)	1 (0.3)	
Pleural adhesions				0.71
Yes, n (%)	206 (32)	88 (30)	118 (32)	
No, n (%)	448 (69)	201 (70)	247 (68)	
Number of Lymphadenectomy Fields				0.64
Two, n (%)	448 (69)	201 (70)	247 (68)	
Three, n (%)	206 (32)	88 (30)	118 (32)	
Abdominal Procedure				<0.001
Hand Assisted Laparoscopic Surgery, n (%)	558 (85)	205 (71)	353 (97)	
Laparotomy, n (%)	96 (15)	84 (29)	12 (3.3)	
Reconstruction Conduit				0.01
Gastric tube, n (%)	621 (95)	272 (94)	349 (96)	
Right colon, n (%)	22 (3.4)	7 (2.4)	15 (4.1)	
Jejunum, n (%)	9 (1.4)	8 (2.8)	1 (0.3)	
Ileum, n (%)	2 (0.3)	2 (0.7)	0 (0)	
Reconstruction Route				0.01
Retrosternal, n (%)	641 (98)	280 (97)	361 (99)	
Posterior mediastinal, n (%)	13 (2.0)	9 (3.1)	4 (1.1)	
Anastomosis Site				0.02
Cervical, n (%)	640 (98)	277 (96)	363 (99)	
Intrathoracic, n (%)	14 (2.1)	12 (4.2)	2 (0.5)	
Conversion to thoracotomy, n (%)	3 (0.5)	1 (0.3)	2 (0.5)	0.70

SD: Standard deviation

**Fig. 3:**
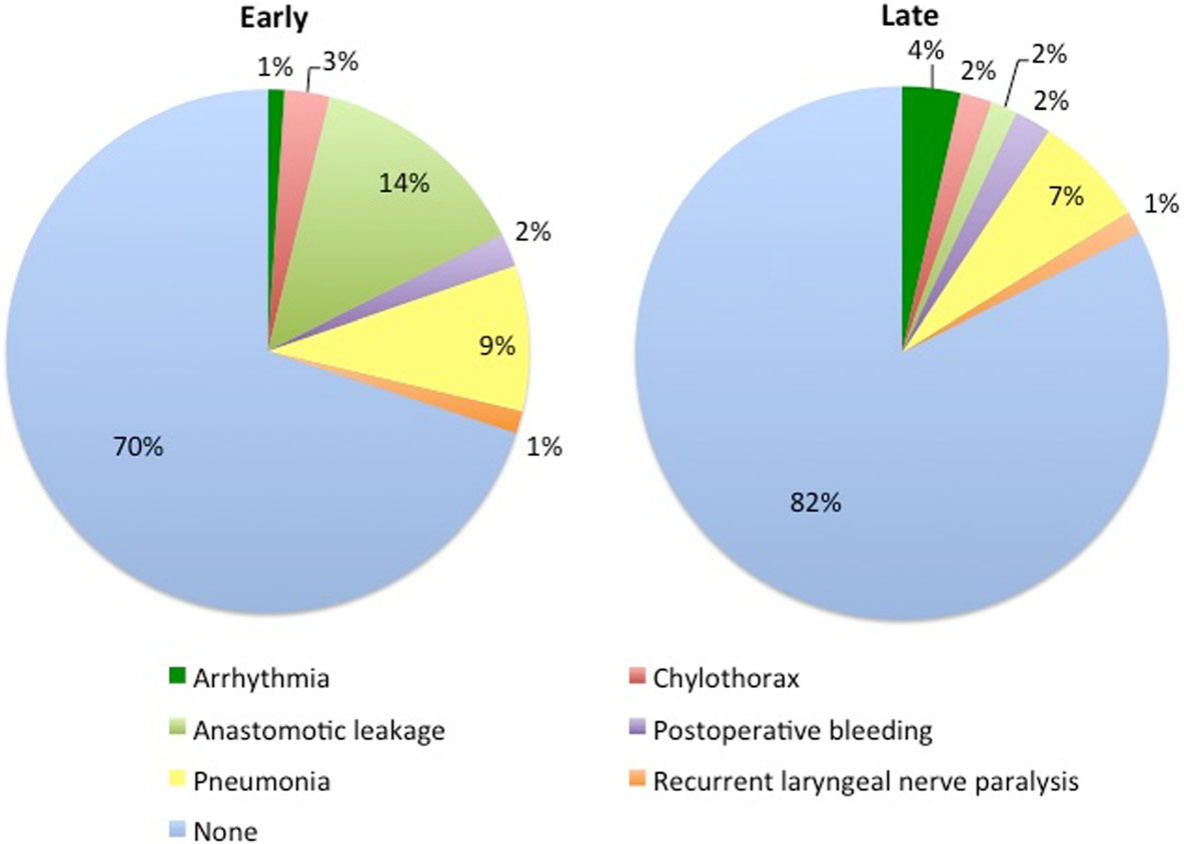
Complications for the two time-periods of the study, Early (left) and Late (right)

The mean age, tumor location, stage, preoperative treatment, abdominal procedure, reconstructed organ, reconstruction route, and anastomosis site significantly differed between the two groups. Surgical outcomes and complications are shown in Table 2 and Fig. 3. Significant differences in surgical outcomes including blood loss, postoperative hospital stay, and number of dissected lymph nodes were found between the two groups. There were no instances of intra-operative complications such as twisting or injury to the gastric tube used for reconstruction.

**Table 2:** Surgical Outcomes and Post Operative Complications

	Early (n = 289)	Late (n = 365)	*p*
Thoracic operative time (min), mean (SD)	210.1 (70.4)	201.1 (64.3)	0.10
Thoracic blood loss (ml), mean (SD)	174.0 (285.0)	94.2 (117.2)	<0.001
Blood transfusion (ml), mean (SD)	108.7 (1346.8)	19.0 (204.5)	0.26
Extubation (days after surgery), mean (SD)	0.19 (1.04)	0.16 (1.73)	0.61
Postoperative length of stay (days), mean (SD)	33.4 (29.8)	20.0 (13.1)	<0.001
Number of retrieved thoracic lymph nodes, mean (SD)	20.0 (13.4)	28.4 (12.1)	<0.001
Number of retrieved total lymph nodes, mean (SD)	43.3 (24.3)	56.1 (24.1)	<0.001
Overall Complications, n (%)	77 (26.6)	47 (12.9)	<0.0001
Surgical Complications			
Chylothorax, n (%)	8 (2.8)	7 (1.9)	0.93
Anastomotic leakage, n (%)	40 (13.9)	6 (1.6)	<0.0001
Postoperative bleeding, n (%)	6 (2.1)	8 (2.2)	0.92
Recurrent laryngeal nerve paralysis, n (%)	4 (1.4)	5 (1.4)	0.99
Non-Surgical Complications			
Arrhythmia, n (%)	3 (1.0)	13 (3.6)	0.04
Pneumonia, n (%)	26 (9.0)	25 (6.8)	0.24
Reoperation within 30 days, n (%)	9 (3.1)	5 (1.4)	0.13
Mortality within 30 days, n (%)	4 (1.4)	0 (0)	0.02

**Fig. 4:**
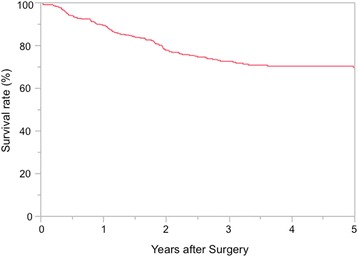
Five-year overall survival for all patients

**Fig. 5:**
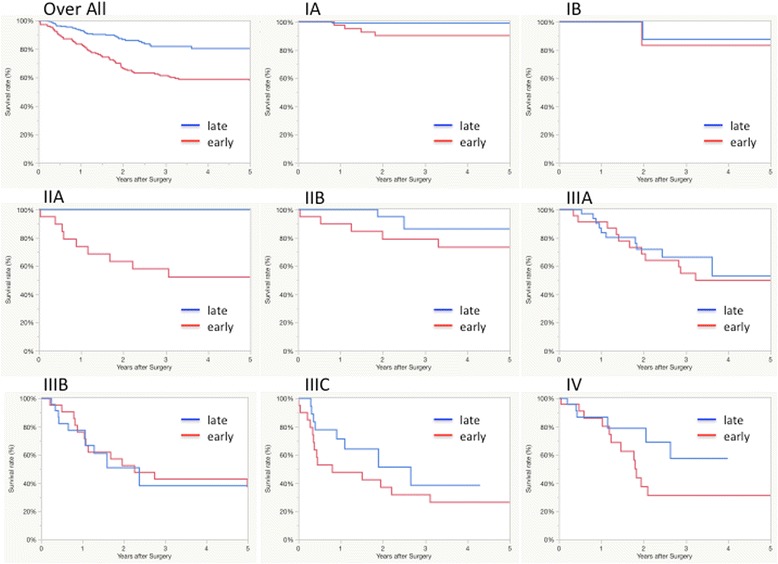
Five-year survival rate by stage for each of the two study time periods, early (red) and late (blue)

There were significant differences in the incidences of overall postoperative complications, postoperative arrhythmias and grade II or higher anastomotic leakage based on the Clavien-Dindo classification. The incidence of arrhythmias was higher in patients treated in the late period, whereas the incidence of anastomotic leak was significantly lower in the late period. The incidence of other complications is not different, comparing the two study time periods. Postoperative mortality in the first 30 days was only seen in the early period 4/654 (0.61%), due to anastomotic leak and pneumonia. Logistic analysis of overall complications in the late period is shown in Table 3 and. The 5-year overall survival, excluding deaths from other diseases, is 70% (Fig. 4), and the 5-year survival rate by stage and each study period are shown in Fig. 5. These data are comparable to data reported by other institutions. [14] Median survival time was analyzed by Log-Rank difference (*P*-value) both overall and at each pathological stage. There is a significant difference in median overall survival (*p* < 0.001), pStage IA (*P* = 0.01) and pStage IIA (P = 0.01). Cox hazard analysis adjusted by operation time, neo adjuvant therapy, blood transfusion, complications showed significantly improved results in the late study period (hazard ratio, 1.72; 95% confidence interval, 1.27–2.32; *p* = 0.00) (Table 4).

**Table 3:** Predictors of Overall Complications in late period

Variables	Odds Ratio	95% Confidence Interval	*p*
Age	1.03	0.99–1.08	0.09
Neoadjuvant therapy	1.12	0.39–4.13	0.83
Abdominal Procedure	0.24	0.04–1.30	0.09
Reconstruction Conduit	0.36	0.04–1.96	0.27
Reconstruction Route	1.11	0.14–6.83	0.35
Anastomosis Site	6.11	0.01–106.2	0.72
Thoracic blood loss	1.00	1.00–1.01	0.06
Number of retrieved thoracic lymph nodes	0.97	0.93–1.01	0.45
Number of retrieved lymph nodes (total)	1.01	0.99–1.03	0.45

**Table 4:** Cox hazard analysis for overall survival

Variables	Hazard Ratio	95% Confidence Interval	*p*
Early	1.00	Reference	
Late	1.72	1.27–2.32	0.00

Adjusted to operation time, neo adjuvant therapy (no adjuvant therapy vs. any adjuvant therapy), blood transfusion (no blood transfusion vs. blood transfusion), complications (no complications vs. any complications)

## Discussion

In Japan, squamous cell carcinoma-derived lesions account for more than 90% of cases of esophageal cancer. Great importance is attached to a thorough lymph node dissection in surgical resection, and open thoracotomy is used as the standard procedure in many institutions, which is highly invasive. In 1992, Cushieri et al. first described the less-invasive thoracoscopic technique for esophageal cancer, [5] and a large-scale, multicenter, prospective study of invasiveness in thoracotomy and thoracoscopic surgery is currently underway in Japan. [6] We performed completely thoracoscopic surgery for esophageal cancer in the left lateral decubitus position on 654 patients between November 1996 and July 2015, representing the largest number of cases of standardized surgery performed in the left lateral decubitus position at a single institution worldwide.

In the early period defined in this study (1996–2008), the surgical procedure was introduced, and surgery was performed mainly by a single operator (M.M). In the late period (2009–2015), the procedure was standardized, and two more operators were trained to perform it (K.O., S.G.). In an evaluation of thoracoscopic surgery in the prone position, Palanivelu et al. found that fewer respiratory complications occurred and that securing ample working space was relatively easy, resulting in shortening of the operative time. [9] The most advantageous aspect of the prone position is that it allows creation of a working space using an artificial pneumothorax with carbon dioxide, and we added this technique to the surgical procedure in the left lateral decubitus position in 2010.

A characteristic of surgery in our institution is the use of a single monitor, which is placed at the head of the operating table, and the visual field axis is set in the direction from the foot of the operating table to the head of the operating table. This arrangement allows lymph node dissection to be advanced parallel to the recurrent laryngeal nerve, and the cervical side can be easily reached, enabling dissection of the lymph nodes around the recurrent laryngeal nerve without distraction. Noshiro et al. found that lymph node dissection around the recurrent laryngeal nerve, performed in the prone position, was comparable to that performed in the left lateral decubitus position. [10] However, this method involves crossing over the trachea, which can result in thermal injury to the trachea by the coagulation device. This method also requires traction on the esophagus. In contrast, when performed in the left lateral decubitus position, the visual field on the ventral side of the left recurrent laryngeal nerve can be easily exposed by only slightly deviating the trachea. Noshiro et al. suggested that although thoracoscopy time was significantly longer (307 ± 66 min vs. 272 ± 58 min, *p* = 0.021), prone esophagectomy had significantly less blood loss (142 ± 87 ml vs. 295 ± 416 ml, *p* = 0.045). However there was no difference in short term outcomes including mortality or perioperative complications. In the present study, blood loss is less than in other reports and we also report a shorter operation time. Feng et al. described the operative time in the chest using the prone position to be shorter than the left lateral position (67 ± 20 min vs. 77 ± 17 min, *p* = 0.013) and number of lymph nodes retrieved is better than when using the left lateral decubitus position (11.6 ± 4.0 vs. 8.9 ± 4.9, *p* = 0.005). [15] These data are not comparable with ours because the number of lymph nodes retrieved is different (present study average is 28.4 ± 4.9 in late study period).

Teshima et al. compared short-term outcomes between the prone and left lateral decubitus positions and there were no significant differences in thoracoscopic surgical time between the groups (247 ± 45 min vs. 236 ± 48 min, *p* = 0.24). [16] Although the surgery was easy to perform in the prone position because of the field of view, this did not lead to shorter procedures. Finally they concluded more time was required when first introducing the method and the operative time gradually decreased. However, thoracic blood loss was significantly lower in the prone position than in the left lateral decubitus position (226 ± 251 g vs. 521 ± 509 g, *p* < 0.01). Our surgical outcomes compare favorably with these data (thoracoscopy time: 201.1 ± 64.3 min, thoracic blood loss: 94.2 ± 117.2 ml). Both positions have some benefits and we believe that the standardized technique in own institution is most important for surgical outcomes.

A significant difference in patient age was observed between patients treated in the early and late study periods. However, no pre-defined age restriction is used in our institution, and activities of daily living and quality of life are first considered in deciding on a treatment strategy. Although active surgical intervention is not generally indicated for older patients in Japan, the oldest patient who underwent this procedure in this series was 93 years of age. This patient greatly benefited from resection, as he was unable to tolerate solid food preoperatively. After surgery, he was discharged on postoperative day 14 and he survived five years after resection, able to care for himself at home.

There were also significant differences in the gender ratio and tumor location, reflecting the retrospective nature of the study, and the tumor stage significantly differed comparing the early and late study periods. Preoperative chemotherapy is now recommended for stage II or III tumors as the standard treatment in Japan, based on the results of the Japanese Clinical Oncology Group trial JCOG9907. [17] Thus, preoperative chemotherapy was administered to only 13% of patients in the early period, but to 82% in the late period, suggesting that downstaging by neoadjuvant therapy caused a significant difference in tumor stage comparing the two study periods. Of particular note are the 62 patients with Stage IV tumors who underwent resection. We were able to achieve an R0 resection in 59 patients, R1 resection in two patients and an R2 resection in one patient. The decision to operate on patients with these advanced lesions is made based on the preoperative CT scan. We believe that neoadjuvant therapy increased the proportion of patients who could undergo an R0 resection, but this requires further study.

We perform hand-assisted laparoscopic resection in the abdomen. This procedure can be rapidly performed and, we believe, allows gentler handling and reconstruction of the stomach. After making a horizontal skin incision, we prepare the greater and lesser omentum sides under direct observation, followed by dissecting the lymph nodes with hand-assisted laparoscopic surgery. This technique shortened the hand-assisted laparoscopic surgery procedure time (to about 20 min), as well as the total operative time. In a study of colorectal resections, Aalbers et al. suggested that hand assisted laparoscopic surgery provides a more efficient segmental colectomy regarding operating time and conversion rate in a systematic review and meta analysis. [18]

We attribute the lack of intraoperative complications such as twisting or injury of the gastric tube to the gentle manipulation of the stomach afforded by the hand-assist technique. In addition, we use nearly the entire stomach for creation of the gastric tube which preserves the blood supply.

Open resection was selected as the first-line treatment for patients with a history of previous laparotomy. In the late study period, hand-assisted laparoscopic surgery was used when possible, even in patients with previous laparotomy. For reconstruction, subtotal gastric tube reconstruction was generally used. In patients with a history of gastric surgery or concomitant gastric cancer, the right colon was used instead. Intestinal reconstruction was performed frequently in the early period but not in the late period. The most commonly used reconstruction route was retrosternal, but the prevalence of patients with previous thoracotomy for heart disease increased in the late period as the indications expanded. In Japan, the retrosternal route was used in 37%, the posterior mediastinal route in 39% and other routes in 24%. [14] Although we used a subtotal gastric tube reconstruction through the retrosternal route, the length of the gastric tube, operation time, and bleeding are all reasonable.

All of these factors may have affected the significant differences in operative data between the time periods. The significant difference in the anastomosis method was due to the selection of intrathoracic anastomosis, using the small intestine for abdominal esophageal and lower esophageal cancers in the early period. In the late period, cervical anastomosis was used in all cases.

An analysis of surgical outcomes is shown in Table 2. There is no significant difference in the intrathoracic operative time. Shortening of the operative time due to improvement of the procedure was expected in the late period. However, operations were performed by surgeons with less experience, and more patients had dense lung adhesions with expansion of the indications for resection in this time period. These factors may explain the absence of a significant change in operative time. In addition, a high definition video system (Endeye HD camera and Visera Elite Video System, Olympus, Tokyo Japan) was introduced in 2012, which permits detailed visualization of the microanatomy and may have made the procedure more delicate, thus prolonging the operative time. However, these refinements also are associated with a significant decrease in blood loss, from 174 mL in the early period to 94.2 mL in the late period, and a significant increase in the number of intrathoracic and total lymph nodes dissected. Thus, the quality of the surgical technique improved in the late period, although there was no change in operative time. As the skills of the three surgeons continue to mature, the goal of shortening the intrathoracic operative time to 2 to 2.5 h may soon be achieved.

The incidence of postoperative complications also significantly decreased in the late period, as shown in Table 4. In 2009, we introduced a standardized postoperative clinical pathway for patients with esophageal cancer, which has permitted standardized postoperative management, with a marked reduction in the incidence of anastomotic leakage from 14% in the early period to 1.6% in the late period. In the late period, patients with diabetes or who had undergone preoperative chemo-radiotherapy were more strictly managed, and the postoperative infusion volume was increased in consideration of the peripheral circulating blood volume. This practice is in contrast to the adjustment of infusion volume to slightly dehydrate in consideration of postoperative circulatory dynamics in the early period. These changes may underlie the marked decrease in the incidence of anastomotic leakage comparing the two study time periods. In contrast, the incidence of arrhythmias significantly increased in the late period, which may be due to the increased numbers of patients with underlying conditions such as heart disease and elderly patients in the late period. The postoperative hospital stay was also markedly shortened in the late period as the complication rate decreased.

In the present study, we investigated all patients who we operated on during a period of 20 years. There were many significant differences between the early and later periods. In the later period, the number of early-stage patients increased in parallel with the increasing rate of preoperative neoadjuvant chemotherapy. In contrast, more patients received neoadjuvant chemo-radiation in the early period than in the later period. The occurrence of anastomotic leakage might be influenced by these differences. In the future, we will perform a prospective study after adjusting for these differences.

There are acknowledged limitations to this study. It is a retrospective study from a single institution, which may limit the general applicability of the technique and results. It is not possible to compare among a variety of surgical techniques with the absence of a control or comparison group, but these data do demonstrate the feasibility and safety of resection in the left lateral decubitus position.

## Conclusions

This analysis of 654 patients treated with thoracoscopic resection of esophageal cancer since 1996 in a single institution shows that a safe, standardized surgical procedure has been established over 20 years, and a training system for new surgeons is successful. The surgical outcomes are satisfactory and possibly superior to those reported by others in some regards. [14–16] This study demonstrates that resection of esophageal cancer in the left lateral position is feasible and safe, using a standardized procedure for thoracoscopic esophagectomy.

## Abbreviations

CT: Computed Tomography
JCOG: Japanese Clinical Oncology Group
